# Comparative analysis of randomized versus real-world populations in heart failure: DIAMOND trial vs CARE-HK registry

**DOI:** 10.1093/eschf/xvag155

**Published:** 2026-05-29

**Authors:** Solenn Fabien, Francesco Fioretti, Victoria Donachie, Achim Obergfell, Javed Butler, Stefan D Anker, Stephen J Greene, Andrew J Sauer

**Affiliations:** CSL Vifor, Flughofstrasse 61, Glattbrugg 8152, Switzerland; Baylor Scott & White Research Institute, Dallas, TX, USA; CSL Vifor, Flughofstrasse 61, Glattbrugg 8152, Switzerland; CSL Vifor, Flughofstrasse 61, Glattbrugg 8152, Switzerland; Baylor Scott & White Research Institute, Dallas, TX, USA; Department of Medicine, University of Mississippi Medical Center, Jackson, MS, USA; Department of Cardiology (CVK) of German Heart Center Charité, German Centre for Cardiovascular Research (DZHK) Partner Site Berlin, Charité Universitätsmedizin, Berlin, Germany; Division of Cardiology, Duke Clinical Research Institute, Durham, NC, USA; Division of Cardiology, Duke University School of Medicine, Durham, NC, USA; Department of Cardiovascular Medicine, Saint Luke’s Mid America Heart Institute and University of Missouri, Kansas City, MO, USA

**Keywords:** Heart failure, Hyperkalaemia, Real-world evidence, Randomized controlled trial, External validity, Clinical trial generalizability

## Abstract

**Introduction:**

The DIAMOND randomized controlled trial (RCT) found patiromer to effectively reduce the risk of hyperkalaemic events among patients with heart failure (HF). However, the extent to which the RCT population reflects routine clinical practice remains unclear. We aimed to compare eligibility, clinical profile, and treatment patterns between patients enrolled in the CARE-HK registry and the DIAMOND trial.

**Methods:**

DIAMOND (NCT03888066), a Phase 3b RCT completed in 2021, and CARE-HK (NCT04864795), a non-interventional registry completed in 2024, both enrolled patients with chronic HF and either active hyperkalaemia or high risk for hyperkalaemia.

**Results:**

Only 17.2% of CARE-HK participants met DIAMOND’s eligibility criteria, dropping to 3.4% when excluding patients with missing data. Compared with DIAMOND, CARE-HK enrolled an older (71.8 vs 67.2 years), more diverse population with more women (31.5% vs 27.6%) and greater burden of comorbidities. Patients exhibited more advanced renal dysfunction, especially in the HF with reduced ejection fraction (HFrEF) population. Within CARE-HK, the use of angiotensin receptor–neprilysin inhibitors (72.1% vs 17.0%), sodium–glucose transport protein 2 inhibitors (69.0% vs 6.6%), and quadruple therapy (47.4% vs 3.9%) was higher in the HFrEF subgroup than in DIAMOND. Despite some progress in prescribing practices, uptake in guideline-directed medical therapy remained incomplete, especially within HFrEF, and renin–angiotensin–aldosterone system inhibitor dosing was often suboptimal.

**Conclusion:**

This study underscores the value of real-world evidence in complementing RCTs by offering insights into population diversity, comorbidity burden, and prescription behaviours, and highlights the need for more pragmatic and inclusive trials in HF.

## Graphical Abstract

**Graphical Abstract:**
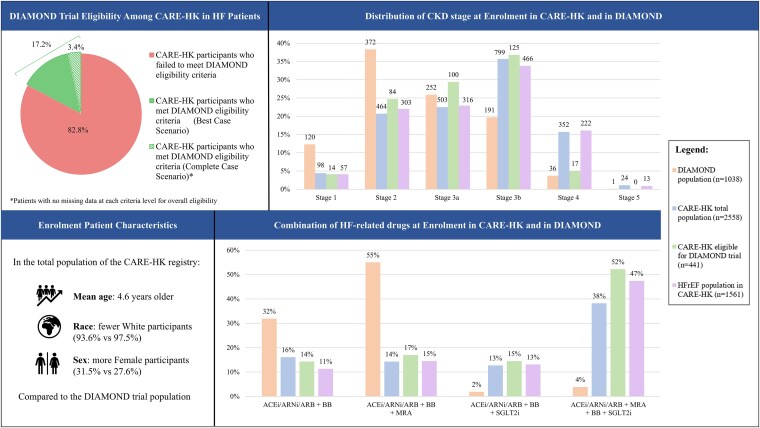

ACEI, angiotensin-converting enzyme inhibitor; ARB, angiotensin receptor blocker; ARNi, angiotensin receptor-neprilysin inhibitor; BB, beta-blocker; CKD, chronic kidney disease; HF, heart failure; HFrEF, heart failure with reduced ejection fraction; MRA, mineralocorticoid receptor antagonist; SGLT2i, sodium-glucose transport protein 2 inhibitors.

## Introduction

Traditional randomized controlled trials (RCTs) are the gold standard for high-quality evidence generation and proving cause–effect relationships.^1,2^ However, in many cases, stringent eligibility criteria and precisely defined clinical environment increase the trial’s internal validity but may compromise external comparability.^1,3^ In cardiovascular RCTs among other therapeutic areas, women, older adults, racial and ethnic minorities, or comorbid patients often remain underrepresented.^4^ Real-world evidence (RWE), derived from real-world data (RWD), may help bridge the evidence gap between efficacy and effectiveness.^5^ The Food and Drug Administration (FDA) defines RWD as routinely collected health data from sources such as claims, registries, patient-reported outcomes, and wearable technologies.^6^ Real-world evidence studies enhance trial population diversity by broadening eligibility and reducing participant burden, promoting equitable research access and public trust.^6,7^ In heart failure (HF), RWE is particularly valuable as hyperkalaemia (HK) is more prevalent among older patients and individuals with comorbidities, such as diabetes or advanced chronic kidney disease (CKD)—populations that are typically underrepresented in clinical trials but constitute the primary target group for potassium binders.^8^

The aim of our study is to compare eligibility, characteristics, and HF therapy patterns between the real-world population enrolled in the CARE-HK (NCT04864795, from 2021 to 2024) registry and the patient population enrolled in the DIAMOND (NCT03888066, from 2019 to 2021) Phase 3b trial to assess real-world generalizability of clinical trial findings. Both studies enrolled patients with chronic HF who either had active HK or were at high risk of developing it and investigated the efficacy and safety of patiromer in managing HK within this population. The temporal and methodological differences between the studies enable an exploration of evolving clinical practices and shifts in guideline adherence over time. We hypothesized that <25% of CARE-HK participants would meet DIAMOND eligibility, that DIAMOND-eligible patients within CARE-HK would be older with more advanced renal disease, and that CARE-HK would show greater use of angiotensin receptor–neprilysin inhibitors (ARNi), sodium–glucose transport protein 2 inhibitors (SGLT2i), and quadruple therapy.

## Methods

### Study populations

DIAMOND was a Phase 3b Double-blind, Placebo-controlled, Randomized Withdrawal, Parallel Group Study, which was completed in 2021.^9,10^ CARE-HK was a non-interventional study (NIS) aiming to reflect the real-world population by observing routine clinical practice and was completed in 2024.^11,12^ In this analysis, the population included patients who completed the run-in phase in DIAMOND (*N* = 1038) along with the entire CARE-HK full analysis set (FAS) (*N* = 2558).

### Eligibility of patients in CARE-HK for DIAMOND

Summaries of CARE-HK patient eligibility for DIAMOND based on the DIAMOND trial inclusion/exclusion criteria were produced. Due to the high level of missing data in the CARE-HK database for assessing the DIAMOND inclusion/exclusion criteria, the overall eligibility was determined based on two assumptions:

1. Assumption 1 (‘Best Case Scenario’): patients with missing data for an individual criterion would meet that DIAMOND criterion and thus be considered for overall eligibility. This approach assumes a ‘best case’ scenario for missing data to allow a meaningful comparison of the two cohorts with regard to demographics, clinical characteristics, and HF treatment patterns at enrolment.
2. Assumption 2 (‘Complete Case Scenario’): patients with no missing data at each criteria level for overall eligibility.

The comparisons performed in this article use the population based on Assumption 1. The population based on Assumption 2 is provided in the eligibility table for completeness.

### Patients characteristics and treatment patterns in CARE-HK and DIAMOND

The DIAMOND population was compared with the following CARE-HK populations and subgroups:

1. CARE-HK FAS: all patients enrolled in the CARE-HK registry who were deemed eligible for inclusion in the FAS (eligibility criteria met, presence of required comorbidity data, no major protocol deviations)
2. DIAMOND-eligible CARE-HK FAS: treating all missing data as ‘Best Case Scenario’, where missingness assumes eligibility for the DIAMOND criteria where it occurs
3. CARE-HK FAS HF with reduced ejection fraction (HFrEF) subgroup: all patients enrolled in CARE-HK who are included in the FAS and have left ventricular ejection fraction (LVEF) categorized as HFrEF at enrolment, regardless of eligibility for the DIAMOND trial

Patient demographics, clinical characteristics, and comorbidities were summarized and compared between the DIAMOND population and the three CARE-HK populations detailed above using χ^2^ tests for categorical variables and a *t*-test for continuous variables, with the exception of *N*-terminal pro-B-type natriuretic peptide (NT-proBNP), where the *P* value is based on the Wilcoxon–Mann–Whitney test. However, this comparison of the study populations and subgroups is not powered to detect differences, and reported *P* values should be interpreted descriptively in this context and treated with caution.

For DIAMOND, enrolment value is defined as the value at screening visit. If this value is not available, the first non-missing value after the first screening date and on or before the first run-in dose is used as the enrolment value. Central laboratory results are included except for potassium. For CARE-HK, enrolment value is defined as the most recent result, or as the closest data within 3 months prior or after the enrolment visit date (+/−91 days), unless otherwise specified. Local laboratory values are included.

In DIAMOND, the informed consent form is signed at the start of the screening visit, whereas in CARE-HK, it is signed at the enrolment visit. For clarity, the screening visit in DIAMOND will be referred to as ‘enrolment’.

The distribution of CKD stage and comorbidities are presented graphically at enrolment in DIAMOND and CARE-HK. Differences between DIAMOND and CARE-HK patients in >50% target dose achieved by individual renin–angiotensin–aldosterone system inhibitor (RAASi) drugs are compared using the chi-squared test. Percentages of guideline-recommended target doses were calculated based on the target doses defined in the CARE-HK analysis.^12^

## Results

### Eligibility of patients in CARE-HK for DIAMOND

DIAMOND trial eligibility criteria were applied to CARE-HK FAS to determine the proportion of patients who would have qualified for the trial. *Table 1* shows that 17.2% of CARE-HK participants met DIAMOND trial eligibility criteria under the ‘Best Case Scenario’—which assumes that patients with missing data for an individual DIAMOND criterion were eligible—and 3.4% under the ‘Complete Case Scenario’, excluding patients with missing data (*Graphical Abstract*). The primary limiting inclusion criteria (IC) were LVEF ≤ 40%, which 33.9% of CARE-HK participants failed to meet, along with the criterion related to the kalemic status, which excluded 37.3% of CARE-HK participants. In the ‘Complete Case Scenario’, enrolment data were missing for 31.9% of patients regarding the New York Heart Association (NYHA) classification and for 54.0% of patients concerning the composite IC, including HF hospitalization, atrial fibrillation, and laboratory values. Notably, only 1.8% of CARE-HK patients from North America were found to be eligible for DIAMOND under this scenario, with 79.3% of them lacking at least one data element required for eligibility assessment.

**Table 1:** DIAMOND trial eligibility among CARE-HK in HF patients

Eligibility criteria	Total population (*N* = 2558)	Europe (*N* = 1726)	North America (*N* = 832)
Subjects eligible for DIAMOND trial^b^	441 (17.2%)	312 (18.1%)	129 (15.5%)
Subjects with missing data	1743 (68.1%)	1083 (62.7%)	660 (79.3%)
Subjects eligible for DIAMOND trial with no missing data^c^	88 (3.4%)	73 (4.2%)	15 (1.8%)
**Inclusion criteria not met**			
1. Informed consent	0	0	0
2. ≥18 years old	0	0	0
3. NYHA Classes II–IV	289 (11.3%)	226 (13.1%)	63 (7.6%)
*Missing*	815 (31.9%)	451 (26.1%)	364 (43.8%)
4. LVEF ≤ 40%	867 (33.9%)	539 (31.2%)	328 (39.4%)
*Missing*	299 (11.7%)	163 (9.4%)	136 (16.3%)
5. Receiving BB for HF or unable to tolerate	432 (16.9%)	277 (16.0%)	155 (18.6%)
6. eGFR ≥ 30 mL/min/1.73 m^2^d^^	276 (10.8%)	216 (12.5%)	60 (7.2%)
*Missing*	317 (12.4%)	143 (8.3%)	174 (20.9%)
7. Hyperkalaemia while receiving ACEi/ARB/ARNI and/or MRA *or* normokalaemia with a history of hyperkalaemia while on RAASi treatment in the last 12 months leading to a subsequent and permanent dose decrease or discontinuation of one or more RAASi medications^e^	953 (37.3%)	569 (33.0%)	384 (46.2%)
*Missing*	373 (14.6%)	193 (11.2%)	180 (21.6%)
8. Female of child-bearing potential must be non-lactating, must have a negative pregnancy test, and must agree to continue using contraception throughout the study and for 4 weeks after study completion	0	0	0
9. *With* hospitalization for HF or equivalent within the last 12 months before enrolment	479 (18.7%)	377 (21.8%)	102 (12.3%)
**(a) *Without* atrial fibrillation at enrolment, BNP^a^ > 150 pg/mL (18 pmol/L) or NT-proBNP > 600 pg/mL (71 pmol/L)** **(b) *With* atrial fibrillation at enrolment, BNP^a^ > 300 pg/mL (35 pmol/L) or NT-proBNP > 1200 pg/mL (142 pmol/L)**			
*or without* hospitalization for HF or equivalent within the last 12 months before enrolment			
**(a) *Without* atrial fibrillation at enrolment, BNP^a^ > 300 pg/mL (35 pmol/L) or NT-proBNP > 1200 pg/mL (142 pmol/L)** **(b) *With* atrial fibrillation at enrolment, BNP^a^ > 600 pg/mL (71 pmol/L) or NT-proBNP >2400 pg/mL (284 pmol/L)**			
*Missing*^f^	1381 (54.0%)	805 (46.6%)	576 (69.2%)
**Exclusion criteria met**			
1. Current acute HF within 4 weeks before enrolment	94 (3.7%)	66 (3.8%)	28 (3.4%)
2. Symptomatic hypotension or systolic blood pressure < 90 mmHg	72 (2.8%)	47 (2.7%)	25 (3.0%)
3. Significant primary aortic or mitral valvular heart disease (except secondary mitral regurgitation due to left ventricular dilatation)	77 (3.0%)	38 (2.2%)	39 (4.7%)
4. Heart transplantation or planned heart transplantation during the study period	1 (<0.1%)	1 (<0.1%)	0
5. Diagnosis of peripartum or chemotherapy-induced cardiomyopathy or acute myocarditis in the previous 12 months	0	0	0
6. Implantation of a cardiac resynchronization therapy device within 4 weeks before enrolment	5 (0.2%)	5 (0.3%)	0
7. Restrictive, constrictive, hypertrophic, or obstructive cardiomyopathy	3 (0.1%)	2 (0.1%)	1 (0.1%)
8. Untreated ventricular arrhythmia with syncope within 4 weeks before enrolment	1 (<0.1%)	1 (<0.1%)	0
9. History of, or current diagnosis of, a severe swallowing disorder, moderate-to-severe gastroparesis, or major gastrointestinal surgery	39 (1.5%)	31 (1.8%)	8 (1.0%)
10. Major CV event within 4 weeks before enrolment	12 (0.5%)	10 (0.6%)	2 (0.2%)
11. Note: criterion included in the new Inclusion Criterion 9	0	0	0
12. Liver enzymes > 5 times upper limit of normal	0	0	0
13. Diagnosis or treatment of a malignancy in the past 2 years, excluding non-melanoma skin cancer and carcinoma *in situ* of the cervix, prostate cancer with Gleason score < 7, or condition highly likely to transform into a malignancy during the study	21 (0.8%)	15 (0.9%)	6 (0.7%)
14. Presence of any condition that places subject at undue risk, prevents complete participation in trial procedure, or potentially jeopardizes data quality	0	0	0
15. Use of any investigational product for an unapproved indication within 4 weeks before enrolment, or currently enrolled in any other type of medical research judged not to be scientifically or medically compatible with this study	0	0	0
16. Hypersensitivity to patiromer or its components	0	0	0
17. Note: criterion modified and partially incorporated in the Exclusion Criteria 18	0	0	0
18. Subjects being treated with or having taken any potassium binder within 7 days before enrolment	365 (14.3%)	218 (12.6%)	147 (17.7%)
19. Employee, spouse, or family member of the sponsor, investigational site or contract research organization	0	0	0

Exclusion criteria # 5, 12, 14, 15, 16, and 19 are not applicable to CARE-HK. A subject can contribute to several criteria.

ACEi, angiotensin-converting enzyme inhibitors; ARBs, angiotensin receptor blockers; ARNi, angiotensin receptor–neprilysin inhibitor; BB, beta-blocker; BNP, brain natriuretic peptide; CV, cardiovascular; eGFR, estimated glomerular filtration rate; HF, heart failure; LVEF, left ventricular ejection fraction; MRA, mineralocorticoid receptor antagonist; NT-proBNP, *N*-terminal pro-B-type BNP; NYHA, New York Heart Association; RAASi, renin–angiotensin–aldosterone system inhibitor.

^a^For subjects treated with ARNi (sacubitril/valsartan) in the previous 4 weeks before enrolment, only NT-proBNP values are to be considered.

^b^Assuming those with missing data would meet eligibility criteria: ‘Best Case Scenario’.

^c^Assuming those with missing data would not meet eligibility criteria: ‘Complete Case Scenario’.

^d^Most recent eGFR at enrolment computed with CKD-EPI formula. Most recent result defined as the data collected at the enrolment visit, or as the closest data within 3 months prior or after the enrolment visit date (+/−91 days).

^e^History of HK based on physician opinion leading to RAASi treatment adjustment. Hyperkalaemia or normokalaemia at enrolment was detected based on laboratory data.

^f^For subjects treated with ARNi, only NT-proBNP values were considered; otherwise, both BNP and NT-proBNP were considered.

### Patients characteristics in CARE-HK and DIAMOND

*Table 2* compares demographics, clinical characteristics, and comorbidities at enrolment between DIAMOND and three CARE-HK cohorts: FAS, DIAMOND-eligible subset, and HFrEF subgroup.

**Table 2:** Patient demographics, clinical characteristics, and comorbidities at enrolment in DIAMOND and in CARE-HK

	DIAMOND Run-in (*n* = 1038)	CARE-HK population					
		Total population (*n* = 2558)		Eligible for DIAMOND trial (*n* = 441)		HFrEF population in CARE-HK (*n* = 1561)	
**Demographics**			*P* value ^a^		*P* value^a^		*P* value^a^
Age (years), *n* (missing)	1038 (0)	2558 (0)		441 (0)		1561 (0)	
*Mean (SD)*	67.2 (10.1)	71.8 (10.8)	***	71.3 (9.4)	***	70.8 (10.4)	***
Region, *n* (%)			***		***		***
*USA/Canada*	77 (7.4%)	832 (32.5%)		129 (29.3%)		435 (27.9%)	
*Latin America*	75 (7.2%)	0		0		0	
*Western Europe and other*	96 (9.2%)	1726 (67.5%)		312 (70.7%)		1126 (72.1%)	
*Central/Eastern Europe*	790 (76.1%)	0		0		0	
*Missing*	0	0		0		0	
Race, *n* (%)			***		***		***
*Black or African American*	19 (1.8%)	98 (4.3%)		13 (3.4%)		64 (4.7%)	
*White*	1012 (97.5%)	2139 (93.6%)		352 (93.1%)		1272 (93.2%)	
*Asian*	0	31 (1.4%)		8 (2.1%)		15 (1.1%)	
*Other*	7 (0.7%)	17 (0.7%)		5 (1.3%)		14 (1.0%)	
*Missing*	0	273		63		196	
Sex, *n* (missing)	1038 (0)	2558 (0)		441 (0)		1561 (0)	
*Female, n (%)*	286 (27.6%)	805 (31.5%)	*	91 (20.6%)	**	363 (23.3%)	*
**Clinical parameters**							
BMI (kg/m^2^), *n* (missing)	1038 (0)	1723 (835)		286 (155)		1048 (513)	
Mean (SD)	28.7 (4.8)	28.8 (6.3)		28.2 (5.4)		28.2 (5.7)	**
Systolic BP (mmHg), *n* (missing)	1038 (0)	2197 (361)		378 (63)		1326 (235)	
*Mean (SD)*	129.5 (14.3)	122.5 (19.8)	***	118.8 (17.2)	***	119.0 (18.4)	***
Heart rate (beats/min), *n* (missing)	1038 (0)	2062 (496)		359 (82)		1240 (321)	
*Mean (SD)*	71.7 (11.1)	70.3 (12.5)	**	70.7 (12.0)		70.0 (12.6)	***
eGFR (mL/min/1.73 m^2^), *n* (missing)	972 (66)	2240 (318)		340 (101)		1377 (184)	
*Mean (SD)*	61.9 (21.0)	48.0 (20.3)	***	52.0 (18.4)	***	48.4 (20.3)	***
Serum creatinine (µmol/L), *n* (missing)	972 (66)	2100 (458)		305 (136)		1287 (274)	
*Mean (SD)*	109.8 (37.1)	136.6 (52.3)	***	126.3 (38.3)	***	138.5 (52.2)	***
NT-proBNP (pg/mL), *n* (missing)	977 (61)	1072 (1486)		117 (324)		732 (829)	
*Median (Q1, Q3)*	1404 (755, 2847)	1401 (548, 3785)		2671 (1628, 5076)	***	1724 (627, 4526)	**
Potassium (mmol/L), *n* (missing)	1000 (38)	2185 (373)		318 (123)		1346 (215)	
*Mean (SD)*	4.98 (0.54)	4.81 (0.61)	***	5.13 (0.51)	***	4.84 (0.61)	***
Potassium ranges, *n* (missing)	1000 (38)	2185 (373)	***	318 (123)	***	1346 (215)	*
≤*5.0 mmol/L*	539 (53.9%)	1332 (61.0%)		92 (28.9%)		794 (59.0%)	
*>5.0–5.5 mmol/l*	347 (34.7%)	633 (29.0%)		177 (55.7%)		409 (30.4%)	
*>5.5–6.0 mmol/l*	79 (7.9%)	182 (8.3%)		42 (13.2%)		115 (8.5%)	
*>6.0–6.5 mmol/l*	24 (2.4%)	30 (1.4%)		6 (1.9%)		22 (1.6%)	
*>6.5 mmol/l*	11 (1.1%)	8 (0.4%)		1 (0.3%)		6 (0.4%)	
History of HK, *n* (%)^b^	961 (92.6%)	1443 (56.4%)	***	299 (67.8%)	***	937 (60.0%)	***
*Missing*	0	0		0		0	
*NYHA functional class, n (missing)*	1037 (1)	1743 (815)	***	290 (151)	***	1108 (453)	***
*Class I*	0	289 (16.6%)		0		163 (14.7%)	
*Class II*	538 (51.9%)	1058 (60.7%)		231 (79.7%)		669 (60.4%)	
*Class III*	491 (47.3%)	376 (21.6%)		58 (20.0%)		261 (23.6%)	
*Class IV*	8 (0.8%)	20 (1.1%)		1 (0.3%)		15 (1.4%)	
LVEF, *n* (missing)^c^	1038 (0)	2551 (7)	***	411 (30)	***	1561 (0)	
*HFrEF (*≤*40%)*	1038 (100.0%)	1561 (61.2%)		411 (100.0%)		1561 (100.0%)	
*HFmrEF (>40%–49%)*	0	386 (15.1%)		0		0	
*HFpEF (*≥*50%)*	0	604 (23.7%)		0		0	

BMI, body mass index; BP, blood pressure; CKD, chronic kidney disease; COPD, chronic obstructive pulmonary disease; eGFR, estimated glomerular filtration rate; HF, heart failure; HFmrEF, heart failure with mildly reduced ejection fraction; HFpEF, heart failure with preserved ejection fraction; HFrEF, heart failure with reduced ejection fraction; HK, hyperkalaemia; LVEF, left ventricular ejection fraction; NT-proBNP, *N*-terminal pro-B-type natriuretic peptide; NYHA, New York Heart Association; SD, standard deviation; USA, United States of America.

^a^ *P* value for categorical variables comes from χ^2^ test. In general, *P* value for continuous variables comes from the *t*-test, with the exception of NT-proBNP, where the *P* value is based on the Wilcoxon–Mann–Whitney test.

^b^For DIAMOND, subjects with a hyperkalaemia diagnosis on the pre-specified medical history case report form that started prior to the first patiromer dose and within 24 months of the first dose. For CARE-HK, this is based on the history of hyperkalaemia based on physician definition Note: history is 24 months prior to enrolment for CARE-HK.

^c^For CARE-HK, reported as the most recent collected in CRF at enrolment visit.

^*^ *P* < .05.

^**^ *P* < .01.

^***^ *P* < .001.

#### Demographics

CARE-HK enrolled an older population than DIAMOND [mean age: 71.8 years (±10.8) vs 67.2 years (±10.1), *P* < .001]. This age difference compared with DIAMOND persisted within the HFrEF subgroup [mean age: 70.8 years (±10.4)] and among CARE-HK participants who met DIAMOND eligibility criteria [mean age: 71.3 years (±9.4)]. Whereas approximately one-third of CARE-HK FAS were female, the proportion declined to one in four within the HFrEF subgroup and further to one in five among the DIAMOND-eligible subset of CARE-HK. These percentages represented notable differences in comparison to the DIAMOND population, which enrolled 27.6% of female participants. The vast majority of participants across cohorts were White (93.1%–97.5%), but the three CARE-HK cohorts included a larger proportion of Black or African American (3.4%–4.7%) and Asian participants (1.1%–2.1%) than DIAMOND (respectively 1.8% and 0%).

#### Clinical characteristics

Mean body mass index was comparable across all cohorts (between 28.2 and 28.8 kg/m^2^), but CARE-HK participants qualifying for DIAMOND exhibited a higher median NT-proBNP compared with the DIAMOND population [respectively 2671 pg/mL (Q1, 1628; Q3, 5076) vs 1404 pg/mL (Q1, 755; Q3, 2847), *P* < .001]. It is, however, important to acknowledge the substantial proportion of CARE-HK patients (58.1% of FAS) with missing NT-proBNP values at enrolment. Left ventricular ejection fraction and NYHA classification were directly influenced by DIAMOND’s eligibility criteria, requiring patients to have HFrEF and fall under NYHA Classes II–IV. Important differences consequently emerged between the CARE-HK FAS and the DIAMOND cohort in terms of LVEF, with 38.8% of CARE-HK patients presenting with either HF with preserved ejection fraction (HFpEF) or HF with mildly reduced ejection fraction (HFmrEF) (online Supplementary Table S1). Despite these differences, the majority of patients across all cohorts were classified as NYHA Class II (60.7% in CARE-HK FAS and 51.9% in DIAMOND), reaching as high as 79.7% among DIAMOND-eligible individuals within CARE-HK. It should, however, be acknowledged that, in the three CARE-HK cohorts, NYHA classification was unavailable for nearly one-third of participants at enrolment.

**Figure 1:**
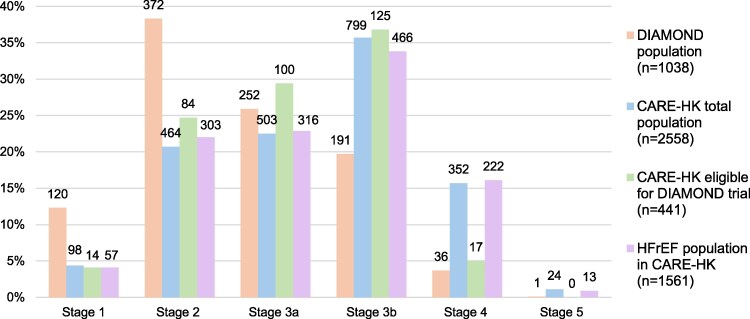
Distribution of CKD stage at enrolment in CARE-HK and in DIAMOND. CKD, chronic kidney disease. For DIAMOND, enrolment value is defined as the value at screening visit. If this value is not available, the first non-missing value after the first screening date and on or before the first run-in dose is used as the enrolment value. For CARE-HK, enrolment value is defined as the most recent result defined as the data collected at the enrolment visit, or as the closest data within 3 months prior or after the enrolment visit date (±91 days), unless otherwise specified

With respect to renal parameters, the estimated glomerular filtration rate (eGFR) was lower (e.g. mean in CARE-HK FAS: 48.0 ± 20.3 mL/min/1.73 m^2^), and the mean serum creatinine was higher (e.g. mean in CARE-HK FAS: 136.6 ± 52.3 µmol/L) at enrolment in the three registry cohorts compared with DIAMOND (mean eGFR, 61.9 ± 21.0 mL/min/1.73 m^2^, and mean serum creatinine, 109.8 ± 37.1 µmol/L). It is important to acknowledge that data on eGFR and serum creatinine were missing for 12.4% and 17.9% of CARE-HK FAS, respectively. *Figure 1* furthermore illustrates the disparities in CKD stages, with 52.5% of CARE-HK FAS diagnosed with CKD Stages 3b–5, compared with only 23.5% of DIAMOND participants. In terms of serum potassium (sK^+^), the mean concentration at enrolment was lower in CARE-HK FAS compared with DIAMOND participants (4.81 mmol/L vs 4.98 mmol/L, *P* < .001). Among CARE-HK participants deemed eligible for DIAMOND, the mean sK^+^ at enrolment was 5.13 mmol/L, with 71.1% of patients presenting with HK (sK^+^ > 5.0 mmol/L). There was also a notable difference in the reported history of HK between CARE-HK FAS and DIAMOND (56.4% vs 92.6%, *P* < .001), although these data were impacted by DIAMOND’s eligibility criteria.

**Figure 2:**
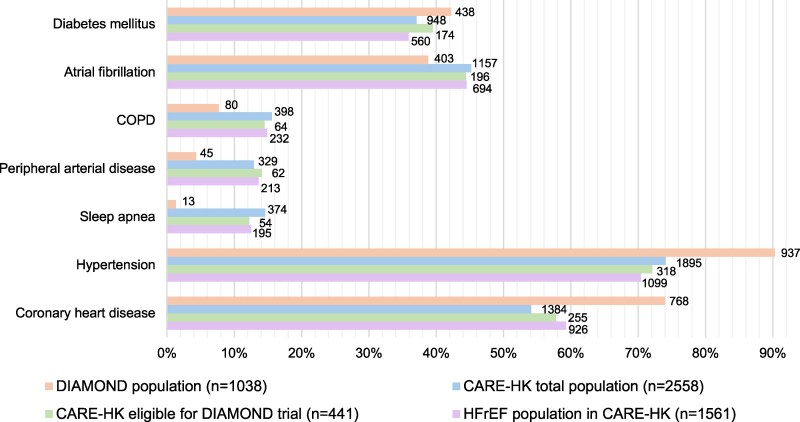
Comorbidities at enrolment in CARE-HK and in DIAMOND. COPD, chronic obstructive pulmonary disease. For DIAMOND, participants with reported history on the pre-specified medical history case report form page. For CARE-HK, patients with comorbidity reported prior or at enrolment using pre-specified categories in the case report form page

Participants in the CARE-HK registry exhibited a lower prevalence of diabetes mellitus, hypertension, and coronary heart disease than those in DIAMOND (respectively 37.1% vs 42.2%, *P* = .004; 74.1% vs 90.3%, *P* < .001; and 54.1% vs 74.0%, *P* < .001) (*Figure 2*). In CARE-HK, the proportions of hypertension were slightly lower (70.4%), and coronary heart diseases were slightly higher (59.3%) among patients with HFrEF compared with the overall enrolled population, although the statistical interpretation of comparisons with the DIAMOND cohort was not impacted. The three registry cohorts exhibited higher prevalences of peripheral arterial disease, chronic obstructive pulmonary disease, sleep apnoea, and atrial fibrillation compared with DIAMOND. At enrolment, 45.2% of CARE-HK FAS notably had atrial fibrillation, while 15.6% had been diagnosed with chronic obstructive pulmonary disease.

### Comparison of treatment patterns

*Table 3* summarizes the use and dosing of RAASi and other concomitant medication for HF treatment at enrolment across DIAMOND and CARE-HK.

**Table 3:** Use and dosing of heart failure medication at enrolment in DIAMOND and in CARE-HK

	DIAMOND Run-in^a^ (*n* = 1038)	CARE-HK population					
		Total population (*n* = 2558)		Eligible for DIAMOND trial (*n* = 441)		HFrEF population in CARE-HK (*n* = 1561)	
**HF treatment prescription, *n* (%)**			*P* value^c^		*P* value^c^		*P* value^c^
ACEi, *n* (missing)	1038 (0)	2558 (0)		441 (0)		1561 (0)	
*Yes, n (%)*	540 (52.0%)	549 (21.5%)	***	72 (16.3%)	***	243 (15.6%)	***
ARB, *n* (missing)	1038 (0)	2558 (0)		441 (0)		1561 (0)	
*Yes, n (%)*	287 (27.6%)	511 (20.0%)	***	46 (10.4%)	***	164 (10.5%)	***
ARNi, *n* (missing)	1038 (0)	2558 (0)		441 (0)		1561 (0)	
*Yes, n (%)*	176 (17.0%)	1446 (56.5%)	***	317 (71.9%)	***	1125 (72.1%)	***
MRA, *n* (missing)	1038 (0)	2558 (0)		441 (0)		1561 (0)	
*Yes, n (%)*	655 (63.1%)	1590 (62.2%)		314 (71.2%)	**	1102 (70.6%)	***
BB, *n* (missing)	1038 (0)	2485 (73)		441 (0)		1527 (34)	
*Yes, n (%)*	998 (96.1%)	2134 (85.9%)	***	441 (100.0%)	***	1381 (90.4%)	***
SGLT2i, *n* (missing)	1038 (0)	2485 (73)		441 (0)		1527 (34)	
*Yes, n (%)*	68 (6.6%)	1503 (60.5%)	***	299 (67.8%)	***	1054 (69.0%)	***
Diuretics, *n* (missing)	1038 (0)	2558 (0)		441 (0)		1561 (0)	
*Yes, n (%)*	997 (96.1%)	2225 (87.0%)	***	393 (89.1%)	***	1401 (89.8%)	***
**HF treatment at ≥50% target dose** ^b^							
ACEi, *n* (missing)	540 (0)	549 (0)		72 (0)		243 (0)	
*Yes, n (%)*	414 (76.7%)	339 (61.7%)	***	44 (61.1%)	**	135 (55.6%)	***
ARB, *n* (missing)	287 (0)	507 (4)		46 (0)		161 (3)	
*Yes, n (%)*	165 (57.5%)	222 (43.8%)	***	15 (32.6%)	**	53 (32.9%)	***
ARNi, *n* (missing)	176 (0)	1442 (4)		316 (1)		1121 (4)	
*Yes, n (%)*	96 (54.5%)	779 (54.0%)		168 (53.2%)		595 (53.1%)	
MRA, *n* (missing)	655 (0)	1587 (3)		314 (0)		1101 (1)	
*Yes, n (%)*	590 (90.1%)	1332 (83.9%)	***	276 (87.9%)		910 (82.7%)	***

ACEI, angiotensin-converting enzyme inhibitor; ARB, angiotensin receptor blocker; ARNi, angiotensin receptor–neprilysin inhibitor; BB, beta-blocker; HF, heart failure; MRA, mineralocorticoid receptor antagonist; SGLT2i, sodium–glucose transport protein 2 inhibitors.

^a^Medication use at date of signing the informed consent form in DIAMOND.

^b^Percentages of guideline-recommended target doses were calculated based on the target doses defined in the CARE-HK analysis.

^c^ *P* value for categorical variables comes from χ^2^ test, and *P* value for continuous variables comes from the *t*-test.

^**^ *P* < .01.

^***^ *P* < .001.

The prescription and dosage of RAASi were compared between the time of informed consent form signature in DIAMOND and CARE-HK. Patients in the CARE-HK FAS were less likely to receive angiotensin-converting enzyme inhibitor (ACEi) (21.5% vs 52.0%, *P* < .001) and angiotensin receptor blocker (ARB) (20.0% vs 27.6%, *P* < .001) compared with those in the DIAMOND population. The ACEi prescription rate notably dropped to 15.6% in the CARE-HK HFrEF subgroup, and among participants from CARE-HK qualifying for DIAMOND, the use of ARBs was as low as 10.4%. In those two drug classes, CARE-HK participants notably received lower doses compared with DIAMOND, with 61.7% of patients being prescribed ≥50% of target dose for ACEi (vs 76.7% in DIAMOND) and 43.8% for ARB (vs 57.5% in DIAMOND). As for ARNi, the proportion of patients receiving such drugs was more than three times larger in the RWE study compared with the Phase 3b trial (56.5% vs 17.0%, *P* < .001). This difference was even more pronounced in the HFrEF subgroup of CARE-HK and among patients meeting DIAMOND eligibility criteria, where ARNi use was over four times higher than in DIAMOND. It is, however, important to note that eligibility for CARE-HK required patients to be prescribed an ACEi, ARB, or ARNi. The prescribed doses were nevertheless comparable, with a majority of patients receiving ≥50% of target dose for ARNi (53.1%–54.5%). Regarding mineralocorticoid receptor antagonist (MRA), prescription rates (62.2%–71.2%) and dosages (82.7%–90.1%) were relatively similar across all cohorts.

**Figure 3:**
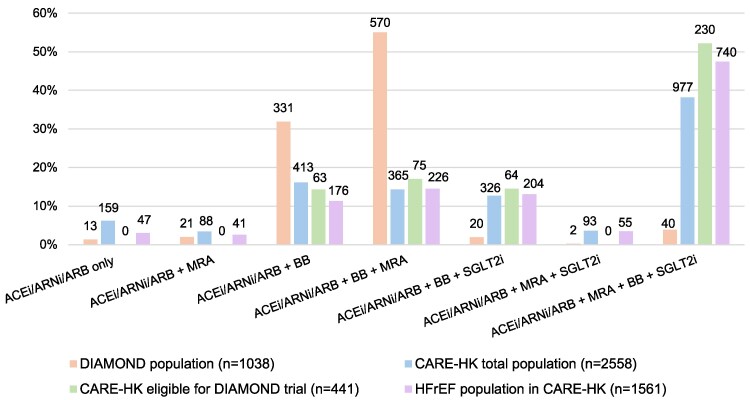
Combination of HF-related drugs at enrolment in CARE-HK and in DIAMOND. ACEI, angiotensin-converting enzyme inhibitor; ARB, angiotensin receptor blocker; ARNi, angiotensin receptor–neprilysin inhibitor; BB, beta-blocker; HF, heart failure; MRA, mineralocorticoid receptor antagonist; SGLT2i, sodium–glucose transport protein 2 inhibitors. Medication use at date of signing the informed consent form in DIAMOND

Regarding HF-concomitant medication, beta-blockers (BBs) were prescribed to over 85% of CARE-HK FAS, 90% of its HFrEF subgroup, and 95% of DIAMOND participants, with BB use being an inclusion criterion in the latter. Diuretics use followed a similar pattern, with prescription rates of 87.0% in CARE-HK FAS, 89.8% in CARE-HK HFrEF subgroup, and 96.1% in DIAMOND. Nevertheless, the use of SGLT2i was considerable higher in the registry (60.5%, *P* < .001) compared with the RCT (6.6%), representing a nine-fold increase. Within the HFrEF subgroup in CARE-HK, SGLT2i prescription rates reached 69.0%, corresponding to a 10-fold difference. Quadruple therapy (ACEi/ARB/ARNi, MRA, BB, and SGLT2i) was the most frequently prescribed treatment in CARE-HK, used in 38.2% of the FAS, 47.4% of its HFrEF subgroup, and 52.2% of the DIAMOND-eligible subgroup. (*Figure 3*) In contrast, 55% of participants from DIAMOND received triple therapy (ACEi/ARNi/ARB, BB, and MRA). Furthermore, only 16.1% of CARE-HK FAS and 11.3% of its HFrEF subgroup received dual therapy (ACEI/ARB/ARNi and BB), compared with 31.9% of DIAMOND patients. Interestingly, there was a substantial regional difference in prescription patterns within CARE-HK (online Supplementary Table S2). Fifty per cent of European participants received quadruple therapy across all HF phenotypes, while this percentage dropped to 16.7% in North America. In the USA, the most frequently prescribed combination was dual therapy (31.9%) followed by triple therapy (19.9%).

## Discussion

In this analysis, 17.2% of participants of CARE-HK met DIAMOND’s eligibility criteria, dropping to 3.4% when excluding patients with missing data. Despite potentially incomplete information, the cohort of 441 patients (17.2%) was retained for comparison with the DIAMOND cohort. This reflects the nature of NISs, relying on routine clinical data and often capturing fewer or less frequent measurements than RCTs. The FAS of CARE-HK enrolled an older, more diverse population with a higher proportion of female and more advanced kidney diseases compared with DIAMOND. Uptake in guideline-directed medical therapy (GDMT) was found to be incomplete in CARE-HK’s HFrEF cohort (47.7%), although SGLT2i use was 10-fold higher (69.0%) than in DIAMOND. Missing data for key parameters (e.g. >50% for NT-proBNP or around 30% for NYHA classification in all CARE-HK cohorts) should be acknowledged as it may account for some of the observed differences between cohorts.

A notably limited overlap was observed between CARE-HK and DIAMOND when assessing the eligibility of CARE-HK patients for inclusion in DIAMOND, which was even more restricted after excluding those with missing data. These findings underscore a critical gap between RCT population and real-world patients—driven by overly stringent eligibility criteria, differences in clinical measurement frequency, and site selection. This highlights the importance of designing clinical trials with broader, more inclusive criteria to better capture the true target population of new therapeutic strategies.^7^

Age and sex differences between CARE-HK FAS and DIAMOND participants were congruent with prior evidence showing underrepresentation of older adults and women in cardiovascular clinical trials.^4,13^ However, female participation remained lower than the real-world prevalence of women affected by HF, since the lifetime risk of developing chronic HF is similar between both sexes.^14^ Strategies such as sex-based enrolment monitoring and adapting eligibility criteria have been proposed to improve female representation in HF trials.^7^ Within CARE-HK, the HFrEF subgroup and the DIAMOND-eligible subset interestingly enrolled a lower proportion of women compared with DIAMOND, possibly due to the lower risk of HFrEF^15^ and lower prevalence of HK in women with HF.^8^ The observed regional differences were largely attributable to site selection. CARE-HK recruited participants from nine countries across Western Europe and the North America, whereas DIAMOND enrolled patients from 21 countries, with the majority coming from Central and Eastern Europe.^10^ Site selection may also have contributed to the broader racial representation observed in the three CARE-HK cohorts compared with DIAMOND, likely reflecting the more heterogeneous demographic composition of the USA. White participants nevertheless remained the majority, while certain minority groups—such as American Indian/Alaska Native or Native Hawaiian/Other Pacific Islander patients—were still underrepresented, even within this real-world research setting.

In the DIAMOND-eligible subset of CARE-HK, the median NT-proBNP value in was twice as high as that observed in DIAMOND, suggesting a greater real-world cardiac burden despite meeting the same eligibility criteria. The higher proportion of DIAMOND-eligible CARE-HK participants presenting with NYHA Class II compared DIAMOND patients aligns with existing evidence suggesting that RCTs tend to enrol healthier patients due to stringent criteria.^16^ This highlights the critical role of RWD in capturing the full clinical heterogeneity of HF populations. Regarding kidney function, CARE-HK’s HFrEF cohort exhibited more severe renal impairment than the DIAMOND cohort, which was evidenced by a lower mean eGFR, a higher mean serum creatinine, and a greater proportion of patients with advanced CKD stages. This difference can be attributed to DIAMOND’s eligibility criteria, which generally excluded patients with severe renal impairment, aligning with previous observations.^7^ Furthermore, the proportion of patients with sK^+^ ≥ 5.0 mmol/L at enrolment within CARE-HK’s HFrEF patients was consistence with prior RWE (39% of HK in HF).^17^ Among DIAMOND-eligible CARE-HK participants, the greater prevalence of HK at enrolment than in DIAMOND likely reflects the variations in eligibility criteria: DIAMOND required patients to have either HK or a history of HK within the last 12 months,^9^ while CARE-HK alternatively included patients with an eGFR of <45 mL/min/1.73 m^2^ or CKD Stage ≥ 3b.^11^ Prevalences of chronic obstructive pulmonary disease observed in CARE-HK and DIAMOND were respectively lower than those reported in epidemiological studies (30%–40%)^18^ and in PARADIGM-HF trial (12.9% in HFrEF).^19^ These discrepancies may stem from the diagnostic complexity of chronic obstructive pulmonary disease, particularly in differentiating it from HF due to overlapping clinical features—leading to frequent under- or misdiagnosis.^18^ The lower proportion of HFrEF patients diagnosed with diabetes and hypertension in CARE-HK than in DIAMOND may be attributed to the broader use of SGLT2i in the registry following recent guideline updates, as these agents help regulate blood sugar levels as well as blood pressure.^20^ Overall, the higher prevalences of sleep apnoea, atrial fibrillation, chronic obstructive pulmonary disease, and peripheral artery disease in the HFrEF population of CARE-HK in comparison to DIAMOND underscores the differences in comorbidity burden between real-world and RCT populations.

Renin–angiotensin–aldosterone system inhibitor therapy (especially MRAs) increases the risk of HK,^21^ often leading to treatment down-titration or discontinuation in patients with HF and/or CKD—thereby limiting the clinical benefit of RAASi agents and worsening cardiorenal outcomes.^22^ However, guidelines emphasize that HK episodes should not preclude RAASi optimization (≥50% of the guideline-recommended target doses for ACEI/ARB/ARNI and MRA) and recommend the use of potassium binder to normalize kalemic levels and support continued therapy.^11^ Angiotensin receptor–neprilysin inhibitor use was about four times higher in the two HFrEF cohorts from CARE-HK compared with the DIAMOND population, accompanied by a two- to three-fold decrease in ARB and ACEi prescriptions when comparing the same populations. This shift likely reflects evolving clinical guidelines, notably the 2022 American Heart Association/American College of Cardiology/Heart Failure Society of America (AHA/ACC/HFSA) and iCARDIO Alliance recommendations of ARNi as first-line therapy for HFrEF,^23,24^ and momentum from the STRONG-HF trial promoting GDMT implementation.^25^ Although European Society of Cardiology (ESC) guidelines still recommend ARNi as a replacement for ACEi,^26^ ARNi prescription rates were unexpectedly higher in Europe than North America, while ACEi and ARB use was lower. These patterns suggests that factors beyond guideline—such as physician preferences, access to medication, or healthcare system differences—may influence treatment implementation in some regions and highlight the need to acknowledge temporal confounding when interpreting the observed differences. As for concomitant HF medication, the lower prescription rates of diuretics in all three CARE-HK cohorts compared with the DIAMOND population may partly reflect the clinical benefit associated with improved implementation of GDMT, which can reduce the need for symptomatic diuretic treatment. Regarding SGLT2i, the 2021 ESC guideline and the 2022 AHA/ACC/HFSA guideline recommended their use in HFrEF patients, whereas in 2023, the ESC extended this recommendation to patients with HFmrEF and HFpEF. ^23,26,27^ While temporal confounding should be acknowledged, the analysis underscores the transformative impact of evolving clinical guidelines and the positive influence of STRONG-HF on the implementation of GDMT,^25^ evidenced by a nine-fold increase of SGLT2i prescription across all HF phenotypes and a 10-fold increase among patients with HFrEF compared with DIAMOND. At the time of DIAMOND, triple therapy—ACEi, ARB, or ARNi combined with a BB and, if needed, an MRA—was the cornerstone of evidence-based treatment for HFrEF,^28,29^ whereas current guidelines now recommend quadruple therapy, adding an SGTL2i to the regimen.^23,26^ Quadruple therapy was more frequently prescribed in CARE-HK, while triple therapy was the most commonly used in DIAMOND. Additionally, a greater proportion of CARE-HK patients received optimal RAASi dosing compared with the CHAMP-HF registry.^30^ These findings suggest encouraging progress in the real-world implementation of guideline recommendations, likely influenced by STRONG-HF,^25^ yet significant gaps remain: half of patients with HFrEF did not receive GDMT, and RAASi doses were commonly suboptimal.

Several limitations should be considered when interpreting these findings. First, the amount of missing data for key variables in CARE-HK—such as NYHA classification (around 30%) and NT-proBNP levels (>50%)—limits the ability to comprehensively characterize the patient profile and may lead to biased or incomplete interpretations of patient eligibility and disease severity. Consequently, the optimistic ‘Best Case Scenario’ assumption should be interpreted with caution, as it may not fully reflect the underlying clinical reality. Second, CARE-HK exclusively used local laboratory data, whereas DIAMOND relied on central laboratories (except for potassium), limiting measurement equivalence and direct comparability. Third, comorbidity prevalence may have been under-estimated; for instance, chronic obstructive pulmonary disease is often underdiagnosed in patients with HF,^18^ and HK history in CARE-HK may be under-reported due to the retrospective data collection and reliance on physician-reported history. Finally, the cross-sectional comparison between DIAMOND (2019–21) and CARE-HK (2021–24) cohorts introduces potential temporal confounding, and differences in site and regional selection may further influence observed therapy patterns.

## Conclusion

This analysis reveals substantial discrepancy between real-world and RCT populations with HF, with CARE-HK participants being older, more diverse, and at markedly higher risk. Treatment differences reflect evolving HF management, despite persistent gaps in RAASi use in HFrEF. The findings underscore the complementary value of RWE in enhancing external validity of RCT findings and emphasize the need for more inclusive trial designs. Future research should compare clinical outcomes across cohorts to assess the real-world impact of GDMT on cardiorenal risk and assess effectiveness of patiromer in HK management and RAASi optimization, using appropriate adjustment methods.

## Supplementary Material

xvag155_Supplementary_Data

## Declarations

### Data Availability

No data were generated or analysed for this manuscript.

### Pre-registered Clinical Trial Number

None supplied.
