# Supplementary material for: Comparative analysis of randomized versus real-world populations in heart failure: DIAMOND trial vs CARE-HK registry
Source: ESC Heart Fail. 2026 May 29;13(3):xvag155. doi: 10.1093/eschf/xvag155 (PMC13281937; doi:10.1093/eschf/xvag155)
Supplement: xvag155_Supplementary_Data [file xvag155_supplementary_data.docx]

**SUPPLEMENTARY MATERIAL**

# Supplementary Table S1. Patient Demographics, Clinical Characteristics and Comorbidities at Enrolment in CARE-HK by LVEF and in DIAMOND

|  | **DIAMOND**  **Run-In**  **(n=1038)** | **CARE-HK population** | | | |
| --- | --- | --- | --- | --- | --- |
|  |  | **HFrEF**  **(n=1561)** | | **HFmrEF**  **(n=386)** | **HFpEF**  **(n=604)** |
| **Demographics** |  |  | p-value [1] |  |  |
| Age (years), n (missing) | 1038 (0) | 1561 (0) |  | 386 (0) | 604 (0) |
| *Mean (SD)* | 67.2 (10.1) | 70.8 (10.4) | *** | 71.4 (11.61) | 74.6 (10.7) |
| Region, n (%) |  |  | *** |  |  |
| *USA/Canada* | 77 (7.4%) | 435 (27.9%) |  | 102 (26.4%) | 295 (48.8%) |
| *Latin America* | 75 (7.2%) | 0 |  | 0 | 0 |
| *Western Europe and Other* | 96 (9.2%) | 1126 (72.1%) |  | 284 (73.6%) | 309 (51.2%) |
| *Central/Eastern Europe* | 790 (76.1%) | 0 |  | 0 | 0 |
| *Missing* | 0 | 0 |  | 0 | 0 |
| Race, n (%) |  |  | *** |  |  |
| *Black or African American* | 19 (1.8%) | 64 (4.7%) |  | 13 (3.7%) | 21 (3.8%) |
| *White* | 1012 (97.5%) | 1272 (93.2%) |  | 337 (95.2%) | 525 (93.8%) |
| *Asian* | 0 | 15 (1.1%) |  | 2 (0.6%) | 13 (2.3%) |
| *Other* | 7 (0.7%) | 14 (1.0%) |  | 2 (0.6%) | 1 (0.2%) |
| *Missing* | 0 | 196 |  | 32 | 44 |
| Sex, n (missing) | 1038 (0) | 1561 (0) |  | 386 (0) | 604 (0) |
| *Female, n (%)* | 286 (27.6%) | 363 (23.3%) | * | 126 (32.6%) | 314 (52.0%) |
| **Clinical parameters** |  |  |  |  |  |
| BMI (kg/m^2^), n (missing) | 1038 (0) | 1048 (513) |  | 252 (134) | 420 (184) |
| Mean (SD) | 28.7 (4.8) | 28.2 (5.7) | ** | 28.7 (6.1) | 30.5 (7.4) |
| Systolic BP (mmHg), n (missing) | 1038 (0) | 1326 (235) |  | 338 (48) | 529 (75) |
| *Mean (SD)* | 129.5 (14.3) | 119.0 (18.4) | *** | 125.3 (20.6) | 129.8 (20.1) |
| Heart rate (beats/min), n (missing) | 1038 (0) | 1240 (321) |  | 314 (72) | 506 (98) |
| *Mean (SD)* | 71.7 (11.1) | 70.0 (12.6) | *** | 70.0 (13.3) | 71.5 (11.8) |
| eGFR (mL/min/1.73m^2^), n (missing) | 972 (66) | 1377 (184) |  | 346 (40) | 510 (94) |
| *Mean (SD)* | 61.9 (21.0) | 48.4 (20.3) | *** | 49.6 (21.9) | 45.6 (18.9) |
| CKD stage, n (%) |  |  | *** |  |  |
| *Stage 1* | 120 (12.3%) | 57 (4.1%) |  | 24 (6.9%) | 17 (3.3%) |
| *Stage 2* | 372 (38.3%) | 303 (22.0%) |  | 70 (20.2%) | 89 (17.5%) |
| *Stage 3a* | 252 (25.9%) | 316 (22.9%) |  | 76 (22.0%) | 110 (21.6%) |
| *Stage 3b* | 191 (19.7%) | 466 (33.8%) |  | 123 (35.5%) | 209 (41.0%) |
| *Stage 4* | 36 (3.7%) | 222 (16.1%) |  | 48 (13.9%) | 79 (15.5%) |
| *Stage 5* | 1 (0.1%) | 13 (0.9%) |  | 5 (1.4%) | 6 (1.2%) |
| *Missing* | 66 | 184 |  | 40 | 94 |
| Serum creatinine (µmol/L), n (missing) | 972 (66) | 1287 (274) |  | 327 (59) | 481 (123) |
| *Mean (SD)* | 109.8 (37.1) | 138.5 (52.2) | *** | 133.6 (52.6) | 133.5 (52.6) |
| NT-proBNP (pg/mL), n (missing) | 977 (61) | 732 (829) |  | 155 (231) | 184 (420) |
| *Median (Q1, Q3)* | 1404 (755, 2847) | 1724 (627, 4526) | ** | 1061 (431, 2466) | 1060 (315, 2397) |
| Potassium (mmol/L), n (missing) | 1000 (38) | 1346 (215) |  | 335 (51) | 497 (107) |
| *Mean (SD)* | 4.98 (0.54) | 4.84 (0.61) | *** | 4.78 (0.61) | 4.73 (0.63) |
| Potassium ranges, n (missing) | 1000 (38) | 1346 (215) | * | 335 (51) | 497 (107) |
| *<=5.0 mmol/L* | 539 (53.9%) | 794 (59.0%) |  | 200 (59.7%) | 333 (67.0%) |
| *>5.0 - 5.5 mmol/l* | 347 (34.7%) | 409 (30.4%) |  | 102 (30.4%) | 120 (24.1%) |
| *>5.5 - 6.0 mmol/l* | 79 (7.9%) | 115 (8.5%) |  | 31 (9.3%) | 36 (7.2%) |
| *>6.0 - 6.5 mmol/l* | 24 (2.4%) | 22 (1.6%) |  | 2 (0.6%) | 6 (1.2%) |
| *>6.5 mmol/l* | 11 (1.1%) | 6 (0.4%) |  | 0 | 2 (0.4%) |
| History of HK, n (%) [2] | 961 (92.6%) | 937 (60.0%) | *** | 226 (58.5%) | 278 (46.0%) |
| *NYHA functional class, n (missing)* | 1037 (1) | 1108 (453) | *** | 262 (124) | 370 (234) |
| *Class I* | 0 | 163 (14.7%) |  | 58 (22.1%) | 68 (18.4%) |
| *Class II* | 538 (51.9%) | 669 (60.4%) |  | 157 (59.9%) | 229 (61.9%) |
| *Class III* | 491 (47.3%) | 261 (23.6%) |  | 46 (17.6%) | 69 (18.6%) |
| *Class IV* | 8 (0.8%) | 15 (1.4%) |  | 1 (0.4%) | 4 (1.1%) |
| **Comorbidities, % [4]** |  |  |  |  |  |
| Diabetes mellitus | 438 (42.2%) | 560 (35.9%) | ** | 133 (34.5%) | 254 (42.1%) |
| Hypertension | 937 (90.3%) | 1099 (70.4%) | *** | 287 (74.4%) | 506 (83.8%) |
| Coronary heart disease | 768 (74.0%) | 926 (59.3%) | *** | 200 (51.8%) | 255 (42.2%) |
| Peripheral arterial disease | 45 (4.3%) | 213 (13.6%) | *** | 43 (11.1%) | 73 (12.1%) |
| Chronic obstructive pulmonary disease | 80 (7.7%) | 232 (14.9%) | *** | 50 (13.0%) | 115 (19.0%) |
| Sleep apnea | 13 (1.3%) | 195 (12.5%) | *** | 49 (12.7%) | 130 (21.5%) |
| Atrial fibrillation | 403 (38.8%) | 694 (44.5%) | ** | 181 (46.9%) | 281 (46.5%) |

BMI, body mass index; BP, blood pressure; CKD, chronic kidney disease; eGFR, estimated glomerular filtration rate; HF, heart failure; HFmrEF, heart failure with mildly reduced ejection fraction; HFpEF, heart failure with preserved ejection fraction; HFrEF, heart failure with reduced ejection fraction; HK, hyperkalaemia; LVEF, left ventricular ejection fraction; NT-proBNP, N-terminal pro b-type natriuretic peptide; NYHA, New York Heart Association; SD, standard deviation; USA, United States of America.

* p < 0.05; ** p < 0.01; *** p < 0.001

[1] P-value for categorical variables comes from chi-squared test. In general, P-value for continuous variables comes from the t-test, with the exception of NT-proBNP where the P-value is based on the Wilcoxon-Mann-Whitney test.

[2] For DIAMOND, subjects with a hyperkalaemia diagnosis on the pre-specified medical history CRF that started prior to first patiromer dose and within 24 months of first dose. For CARE-HK, this is based on history of hyperkalaemia based on physician definition Note: history is 24 months prior to enrolment for CARE-HK.

[3] For CARE-HK, reported as the most recent collected in CRF at enrolment visit.

[4] For DIAMOND, subjects with reported history on the pre-specified medical history CRF page. For CARE-HK, patients with comorbidity reported prior or at baseline using pre-specified categories in the CRF page.

# Supplementary Table S2. Use and Dosing of HF-medication at Enrolment in Patients with at least one HF Medication in CARE-HK by region

|  | **Total population**  **(N=2558)** | **Europe**  **(N=1726)** | **North America**  **(N=832)** |
| --- | --- | --- | --- |
| **HF treatment prescription, n (%)** |  |  |  |
| ACEi, n (%) | 549 (21.5%) | 346 (20.0%) | 203 (24.4%) |
| ARB, n (%) | 507 (19.8%) | 262 (15.2%) | 245 (29.4%) |
| ARNi, n (%) | 1446 (56.5%) | 1064 (61.6%) | 382 (45.9%) |
| MRA, n (%) | 1590 (62.2%) | 1227 (71.1%) | 363 (43.6%) |
| BB, n (%) | 2134 (85.9%) | 1457 (87.5%) | 677 (82.6%) |
| SGLT2i, n (%) | 1503 (60.5%) | 1221 (73.3%) | 282 (34.4%) |
| Diuretics, n (%) | 1612 (64.9%) | 1026 (61.6%) | 586 (71.5%) |
| **Combination of HF-related drugs** |  |  |  |
| ACEi/ARNi/ARB only | 120 (4.8%) | 40 (2.4%) | 80 (9.7%) |
| ACEi/ARNi/ARB + MRA | 55 (2.2%) | 31 (1.8%) | 24 (2.9%) |
| ACEi/ARNi/ARB + BB | 413 (16.6%) | 151 (9.0%) | 262 (31.9%) |
| ACEi/ARNi/ARB + BB + MRA | 363 (14.6%) | 200 (12.0%) | 163 (19.9%) |
| ACEi/ARNi/ARB + BB + SGLT2i | 326 (13.1%) | 220 (13.2%) | 106 (12.9%) |
| ACEi/ARNi/ARB + MRA + SGLT2i | 93 (3.7%) | 69 (4.1%) | 24 (2.9%) |
| ACEi/ARNi/ARB + MRA + BB + SGLT2i | 970 (39.0%) | 833 (50.0%) | 137 (16.7%) |
| Missing | 73 | 61 | 12 |

ACEI, angiotensin-converting enzyme inhibitor; ARB, angiotensin receptor blocker; ARNi, angiotensin receptor-neprilysin inhibitor; BB, beta-blocker; HF, heart failure; MRA, mineralocorticoid receptor antagonist; SGLT2i, sodium-glucose transport protein 2 inhibitors.
